# Distal [FeS]-Cluster Coordination in [NiFe]-Hydrogenase Facilitates Intermolecular Electron Transfer

**DOI:** 10.3390/ijms18010100

**Published:** 2017-01-05

**Authors:** Alexander Petrenko, Matthias Stein

**Affiliations:** Max Planck Institute for Dynamics of Complex Technical Systems, Molecular Simulations and Design Group, Sandtorstrasse 1, 39106 Magdeburg, Germany; petrenko@mpi-magdeburg.mpg.de

**Keywords:** electron transfer, Marcus equation, enzymatic fuel cell, hydrogen oxidation, electrode adsorption, DFT, bioelectrochemistry

## Abstract

Biohydrogen is a versatile energy carrier for the generation of electric energy from renewable sources. Hydrogenases can be used in enzymatic fuel cells to oxidize dihydrogen. The rate of electron transfer (ET) at the anodic side between the [NiFe]-hydrogenase enzyme distal iron–sulfur cluster and the electrode surface can be described by the Marcus equation. All parameters for the Marcus equation are accessible from Density Functional Theory (DFT) calculations. The distal cubane FeS-cluster has a three-cysteine and one-histidine coordination [Fe_4_S_4_](His)(Cys)_3_ first ligation sphere. The reorganization energy (inner- and outer-sphere) is almost unchanged upon a histidine-to-cysteine substitution. Differences in rates of electron transfer between the wild-type enzyme and an all-cysteine mutant can be rationalized by a diminished electronic coupling between the donor and acceptor molecules in the [Fe_4_S_4_](Cys)_4_ case. The fast and efficient electron transfer from the distal iron–sulfur cluster is realized by a fine-tuned protein environment, which facilitates the flow of electrons. This study enables the design and control of electron transfer rates and pathways by protein engineering.

## 1. Introduction

Hydrogen is one of the future energy carriers [1]. It can be used to produce higher energy-rich biofuels or directly for combustion in a turbine or used in a fuel cell. Low-cost, low-carbon (e.g., CO_2_-emitting) hydrogen production can be realized from electrolytic production of dihydrogen using sustainable sources of electricity or from water splitting using sunlight by artificial photosynthesis. In the longer term, vehicle driving can be obtained from electricity, hydrogen or a combination of both in a fuel cell.

Microbial fuel cells (MFCs) are bio-electrochemical converters of microbial-reducing power (generated by the metabolism of substrate), into an electric current by making use of enzymes. Only membrane-less biological fuel cells (FCs) make use of direct electron transfer (DET) reactions to the anode and from the cathode.

Uptake [NiFe] hydrogenase enzymes, which catalyze the reversible oxidation of H_2_, require a fast separation of proton and electron transfer pathways in order to avoid a recombination reaction.

(1) $$H_{2}⇄H^{+}+H^{-}⇄{2H}^{+}+{2e}^{-}$$

**Figure 1:**
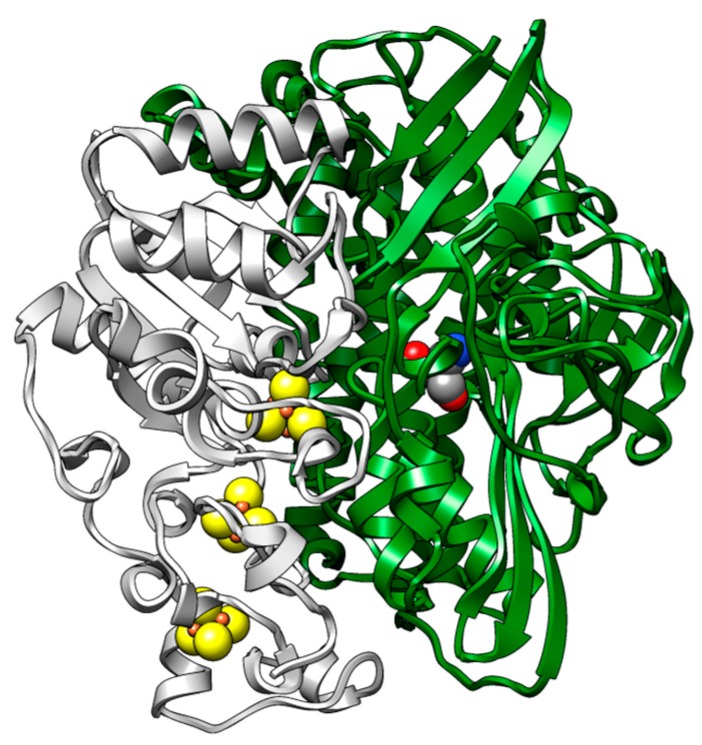
Spatial arrangement of metallic cofactors in [NiFe]-hydrogenase enzyme. The active site (grey, red and blue space-fill model) is in the large subunit (green), a chain of FeS-clusters (yellow) is located in the small subunit (grey).

While the involvement of a glutamate residue in proximity to one of the terminal cysteine residues has been established as a gateway for proton transfer, see [2] for recent spectroscopic and [3] for structural evidence, the routes for electron transfer are less studied. The arrangement of three FeS-cluster in the small subunit, however, suggests the flow of electrons along these metallic cofactors (see Figure 1). The electron transfer path of the [NiFe]-hydrogenase in the small subunit is made up of aligned iron–sulfur clusters, the proximal [4Fe–4S]-, the medial [3Fe–4S]-, and the distal [4Fe–4S]-cluster, which can be characterized by protein film voltammetry, see [4,5] for review articles.

The distal [4Fe4S]_d_ has an unusual three-cysteine and one-histidine coordination, a motif that also occurs in other redox active enzymes such as nitrate reductases and ubiquinone oxidoreductases.

The fast and efficient electron transfer to an electrode surface points to a highly engineered protein environment that modulates both catalysis and proton and electron transfer. We have previously used Brownian Dynamics (BD) simulations to sample the hydrogenase orientation on a graphite electrode surface [5,6]. Residues Ser196, Glu461 and Glu464 were found to establish contacts between the *D. fructosovorans* [NiFe]-hydrogenase and graphite surface. Different electron transfer routes connecting the distal FeS-cluster and the graphite electrode were investigated quantum mechanically. Only one suggested electron transfer pathway via Phe193 to residue Ser196 was found to yield electron transfer rates in excellent agreement with experiments.

Here, we extend this approach to investigating the effect of a histidine-to-cysteine backmutation in the distal FeS-cluster to recover a standard four-cysteine [Fe_4_S_4_]-cubane coordination and show its effect on electron transfer rates. Substitution of a His-to-Cys does not drastically change the reorganization energy of the distal FeS-cluster. Rather, the electronic coupling matrix of the Marcus equation is diminished due to a reduced atomic overlap. This affects the electron transfer rates and reduces them by about three orders of magnitude, which is in excellent agreement with experiment.

## 2. Results and Discussion

**Figure 2:**
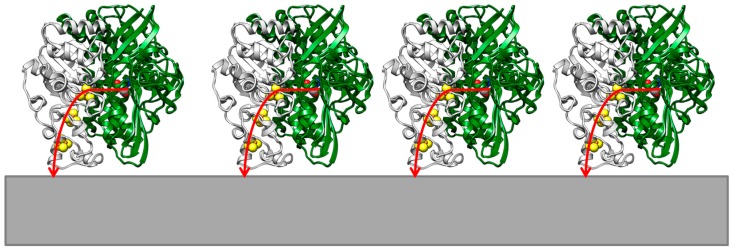
Schematic display of the orientation of hydrogenase enzyme on a graphite electrode. The flow of electrons from the active site (blue, red, grey space-fill representation), via the FeS-clusters (yellow) to the graphite electrode (grey) is indicated.

The nickel–iron hydrogenase absorbs directly on a graphite electrode [6] and also on carbon-nanotube-coated pyrolytic graphite electrodes [7] to, here, catalyze the consumption of H_2_ (see Figure 2). When adsorbed on an electrode, the hydrogenase enzyme produces high catalytic current and occurs at potentials expected for this half-cell reaction [2]. Turn-over-frequencies (TOFs) of up to 50 × 10^4^ s^−1^ have been measured on electrodes.

The activity of the adsorbed enzyme is greater than the catalytic activity with electron acceptors and donors [6]. This demonstrates that both the reaction with H_2_ and electron transfer are very efficient and do not require any overpotential as excessive driving force. The catalytic rate is thus only diffusion-controlled by H_2_ approach to the active site [8].

In the absence of an overpotential, the Marcus equation for electron transfer applies. In the high temperature limit, the rate constant for long-distance electron transfer from a donor to an acceptor is given by the Marcus equation:

(2) $$k_{ET}=\frac{2\pi }{\hbar }{\left|V_{DA}\right|}^{2}{\frac{1}{\sqrt{4\pi \lambda k_{B}T}}}^{\left[-\frac{{\left(\Delta G^{0}+\lambda \right)}^{2}}{4\mu k_{B}T}\right]}$$

where *ħ* is the reduced Planck constant, *k*_B_ the Boltzmann constant and *T* the temperature. The rate is then determined by the electronic coupling matrix element (*V_DA_*), the overall Gibbs free energy change of the electron transfer (ET) reaction (Δ*G*^0^) and the reorganization energy (*λ*) needed by the system to adapt to its new state after electron transfer.

We used the Linderberg type formula for electron transfer to calculate the electronic coupling matrix between interacting donor and acceptor molecules.

(3) $$V_{DA}=\frac{1}{Z}\frac{\partial S}{\partial Z}$$

where *Z* is the distance between the centers of gravity of the electron clouds representing the distribution of the transferring electron in the initial and in the final states. *S* is the electronic overlap integral of the wave functions of electron in its initial (*ψ_a_*) and final (*ψ_b_*) states:

(4) $$S=∭\psi_{a}\left(r\right)\psi_{b}\left(r\right)dxdydz$$

The wave functions of the electron in its initial (*ψ_a_*) and final (*ψ_b_*) states are given as linear combinations of Gaussian basis functions.

The reorganization energy *λ* in the Marcus equation describes the need of the system to adapt to its new electronic state after ET. The reorganization energy has two components, the internal (*λ_i_*) and solvent (*λ_s_*) reorganization energy:

(5) $$\lambda =\lambda_{i}+\lambda_{s}$$

The internal reorganization energies *λ_i_* were computed using separate fragments by means of the Nelsen four-point method [9]

(6) $$\lambda_{i}=E(D^{+}|D)\;-E(D^{+}|D^{+})\;+E(A^{-}|A)\;-E(A^{-}|A^{-})$$

where *E*(*D*^+^|*D*) denotes the total energy of a donor molecule in its oxidized state at the geometry of reduced state, *E*(*D*^+^|*D*^+^) denotes the total energy of a donor molecule in its oxidized state at the geometry of oxidized state, *E*(*A*^−^|*A*) denotes the total energy of the acceptor molecule in its reduced state at the geometry of oxidized state, *E*(*D*^+^|*D*^+^) denotes the total energy of the acceptor molecule in its reduced state at the geometry of reduced state.

Table 1 gives the calculated inner sphere reorganization energies of cluster models of the distal [Fe_4_S_4_](His)(Cys)_3_^−2/−1^ and substituted four-cysteine coordinated [Fe_4_S_4_](Cys)_4_^−3/−2^. Histidine coordination does not significantly influence the reorganization energy and the calculated values agree with those from Sigfridsson et al., who also used the B3LYP exchange-correlation functional with a slightly enhanced double-zeta plus polarization (DZP) basis set [10]. According to these results, the reorganization energy is not a major factor between histidine- and cysteine-coordinated cubanoid FeS-clusters.

The experimental feasibility of changing the first ligand coordination sphere of the distal [FeS]-cluster was demonstrated. Dementin et al. [11] have shown that protein cluster assembly, protein stability as well as active site catalytic function are not affected by histidine-to-cysteine or glycine mutations directly coordinating the distal [FeS]_d_-cluster. Intramolecular ET, however, was impaired by the H184G, and intermolecular ET, by the H184C mutant.

The solvent reorganization energy *λ_s_* was calculated following the procedure described in [12] where *λ_s_* is obtained as the energy difference between the calculated internal *λ_i_* values of the solvated molecular system calculated (in the framework of dielectric continuum solvation model) in two continuum solvents, one (*λ*′) corresponding to the four-point method calculation using the static dielectric constant of solvent, and the other (*λ*′′) using the optical dielectric constant of the solvent:

(7) $$\lambda_{s}=\lambda \prime -\;\lambda \prime \prime$$

In addition to the inner sphere reorganization energy, the solvent reorganization energy has to be considered additionally. Table 2 gives total internal reorganization energies when solvent effects are incorporated.

Effects of the environment on the reorganization energy can be approximated by COSMO calculations of the donor and acceptor models, respectively.

Electron transfer from an iron–sulfur cluster model to a neutral coronene acceptor is associated with an internal reorganization energy of 0.45 eV in vacuo and 0.51 eV when solvent effects are incorporated. This is in agreement with the calculated outer-sphere reorganization energy for the electron transfer between two iron–sulfur cubane [4Fe–4S] clusters in ferredoxin [13] and we can thus transfer the calculated reorganization energies from His-to-Cys coordinated clusters.

**Figure 3:**
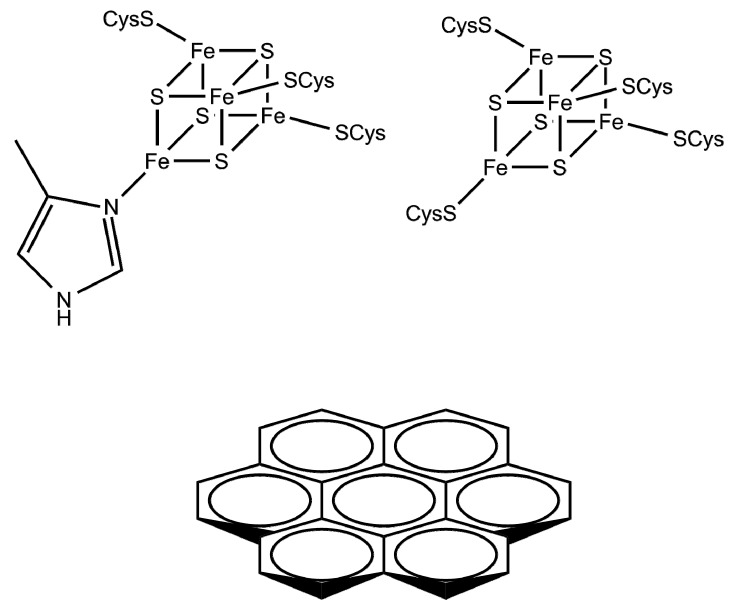
Small cluster models for direct electron transfer from the [FeS]_d_-cluster to a graphite surface model (here coronene).

We have previously shown that the overlap integrals, the electronic coupling matrix V_DA_ and the resulting rates of electron transfer *k_ET_* are almost independent of the spatial extension of the acceptor model (coronene C_24_H_12_ vs. circumcoronene C_54_H_18_), and also the relative orientation of the donor (basal vs. edge) [14]. For reasons of computational efficiency, we have here thus chosen a basal orientation of the coronene with respect to the distal [FeS]-cluster models (see Figure 3).

For [NiFe]-hydrogenase absorbed on a graphite electrode, direct interfacial electron transfer is fast and independent to the presence or absence of a mediator [15]. This indicates (i) a direct contact between enzyme and electrode surface so that mediator molecules cannot interfere with electron transfer; (ii) the distal iron–sulfur cluster [FeS]_d_ in close proximity to the electron acceptor; and (iii) a uniform distribution of enzymes on the electrode surface (see Figure 2). When using an artificial soluble electron acceptor (methyl viologen), the measured rates of electron transfer are even slower than those of electrode-associated [NiFe]-hydrogenases [6].

When we calculate the rates of direct electron transfer *k_ET_* for small cluster models (see Figure 3) using the Marcus equation (2), parameters and integrals defined in equations for *V_DA_* (3), overlap integrals *S* (4) and reorganization energy *λ* (5), we see a striking difference in histidine- vs. cysteine-coordinated distal FeS-clusters (see Table 3).

The calculated rate of electron transfer from the histidine-coordinated distal [FeS]_d_-cluster using the Marcus equation of 5100 s^−1^ is in good agreement with experimental results from Dementin et al. [16] (1050–3100 s^−1^) and Pershad et al. [6] (1500–9000 s^−1^). When the coordinating histidine is replaced by a cysteine residue, the electron transfer slows down by orders of magnitude (to 6.0 s^−1^) due to a significant decrease of electronic coupling between the iron–sulfur cluster and the electrode (see Table 3). Due to the quadratic dependence of electron transfer rate constants on the electronic coupling in the Marcus equation, the ET rate constants differ from 6 to 5100 s^−1^.

Brownian Dynamics simulations have been used to efficiently sample the protein orientation at the graphite surface and identify amino acid residues which mediate the flow of electrons from the distal [FeS]-cluster to the electrode. Terminal residues Ser196, Glu461 and Glu464 were shown to approach the surface, but only the pathway from [FeS]_d_ to the alpha-helical terminus residue Ser196 gave ET rates in good agreement with experiment [14]. Routes leading to Glu461 and Glu464 could be ruled out due to rates of ET orders of magnitude deviating from experiment. The ET is equally efficient via a through-bond (mediated by the peptide main chain) or a through-space ET (mediated by the amino acid side chains) path (2.2 vs. 5.8 × 10^3^ s^−1^).

**Figure 4:**
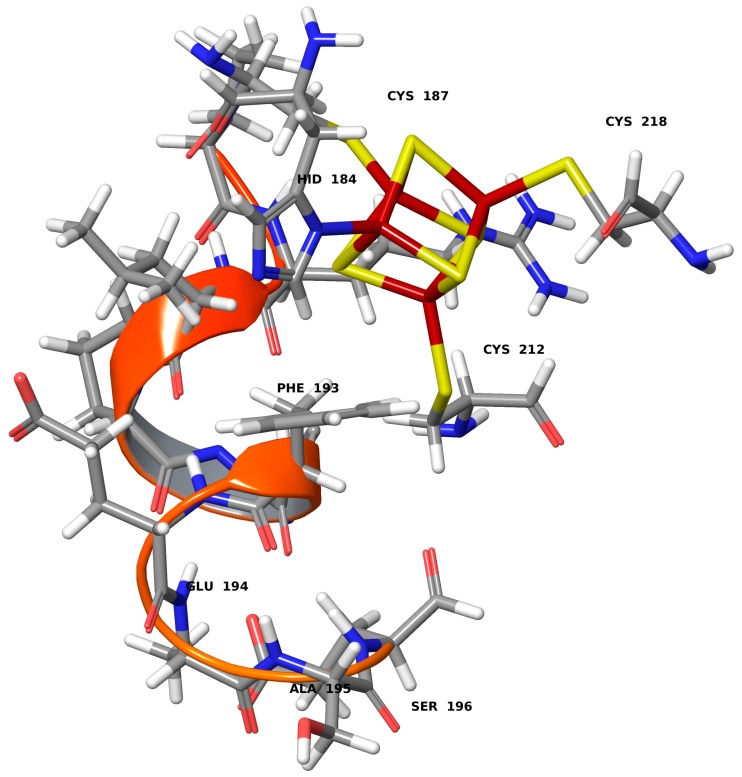
Large structural model for protein-mediated electron transfer from the [FeS]_d_-cluster via Ser196 to an electrode.

Table 4 gives the calculated electron transfer rates from the distal [FeS]_d_-cluster in either a histidine or an all-cysteine coordination sphere via amino acid residues along the preferred route D^185^-S^196^ (Asp185, Asn186, Cys187, Pro188, Arg189, Leu190, Pro191, His192, Phe193, Glu194, Ala195, Ser196) (see Figure 4). This is the shortest path connecting the distal FeS-cluster with the electrode approaching Ser196 residue (via a short α-helical stretch from residues 185–194).

Electrochemical investigation of the wild-type and an all-cysteine coordinated hydrogenase mutant gave significantly reduced rates of electron transfer from the distal [FeS]_d_-cluster to the electrode [11]. Our calculated value of 360 s^−1^ is in excellent agreement with the experimental results of >40 s^−1^.

In general, the DET rates from the distal [FeS]-cluster to the electron acceptor slightly deviate from the measured rates of electron transfer. Calculated values of 5100 and 6 s^−1^ compare well with experimental results of 1050–3500 and 40 s^−1^, respectively. The reproduction of such a subtle experimental effect from sole quantum chemical calculations alone is very encouraging. The slight deviation can be rationalized by the complete neglect of the protein environment. When the electron transfer route via amino acid residues from the [FeS]_d_-cluster to the terminal residue is explicitly incorporated, the calculated rates of electron transfer of 2200 and 360 s^−1^ are in perfect agreement with experiment.

From our calculations, we can thus estimate the effect of the highly sophisticated protein environment for electron transfer from the distal [FeS]-cluster to an electron acceptor (cytochrome or electrode) by comparing Table 2 and Table 3. The first ligation sphere (histidine vs. cysteine) is responsible for the dominating effect on ET rates. The reorganization energy for a [Fe_4_S_4_](Cys)_4_-cubane cluster, however, is almost indistinguishable from that of the distal cluster [Fe_4_S_4_](His)(Cys)_3_ coordination. Clearly, it is not the reorganization energy that is causing the difference in ET rates.

The different electronic coupling between the amino acid histidine with its π-electrons and the aromatic amino acid phenylalanine Phe193 in close spatial contact facilitate a swift through-space transfer of electrons (see Figure 4). Such an interaction is absent in an all-cysteine coordinated distal cubane cluster. The electron transfer rate along the amino acids connecting the distal [FeS]-cluster of hydrogenase with its electron acceptor is equally as fast and facile as the DET. The rates *k_ET_* only marginally change, which shows an efficient protein-mediated electron transfer pathway. The protein environment of the distal cluster is then not only a protectant of the redox active transition metal cluster, but also a highly evolved electron transfer path maintaining an efficient ET.

**Figure 5:**
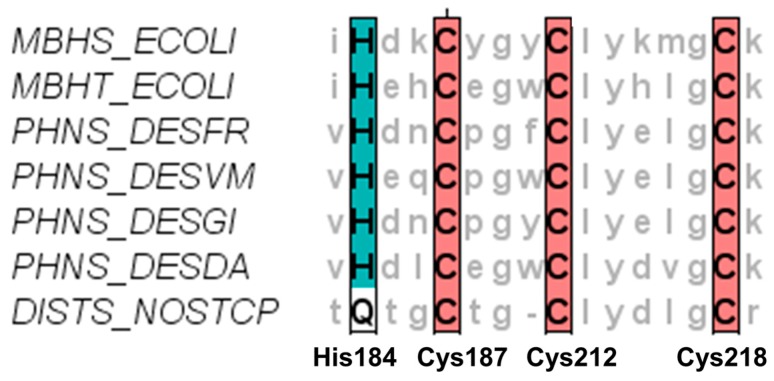
First shell amino acid coordination sphere of the distal FeS-cluster of the small subunit of [NiFe]-hydrogenases (three cysteines (C) 187, 212 and 218 brown, one histidine (H) 184 turquoise, or alternatively, a glutamine (Q) white at that position): *MBHS_ECOLI* (hydrogenase-1; hyaA; *E. coli*), *MBHT_ECOLI* (hydrogenase-2; hybO; *E. coli*), PHNS_DSEFR (periplasmic [NiFe] hydrogenase small subunit; hydA; *D. fructosovorans*), PHNS_DESVM (periplasmic [NiFe] hydrogenase small subunit; hydA; *D. vulgaris*), PHNS_DESGI (periplasmic [NiFe] hydrogenase small subunit; hydA; *D. gigas*), PHNS_DESDA (periplasmic [NiFe] hydrogenase small subunit; hydA; *D. desulfuricans*), DIST_NOSTCP (Ni,Fe-hydrogenase I small subunit; Nos7524_1174; *Nostoc* sp.).

Sequence analysis of amino acid residues in the first coordination sphere of the distal [FeS]-cluster of a representative subset of [NiFe]-hydrogenases is given in Figure 5. All hydrogen-oxidizing [NiFe]-hydrogenases from membrane-bound *E. coli*, or periplasmic hydrogenases from *D. fructosovorans*, *D. vulgaris*, *D. gigas* and *D. desulfuricans*, display the characteristic His184, Cys187, Cys212 and Cys218 coordination (in *D. fructosovorans* amino acid numbering). They are thus expected to possess identical reorganization energies λ and similar direct electron transfer rates from the distal the [FeS]_d_-cluster to a graphite electrode surface. Only differences in amino acid residues spatially connecting [FeS]_d_ and the electrode could slightly modulate the protein-mediated ET rates. The distal [FeS]-cluster of the cyanobacterial uptake [NiFe]-hydrogenase from *Nostoc* sp. [17] (strain ATCC 29411/PCC 7524) and other cyanobacterial hydrogenases [18], however, are missing the bacterial histidine coordination and possess a glutamine at that position. Raleiras et al. have characterized the iron–sulfur clusters of *Nostoc punctiforme* using electron paramagnetic resonance (EPR) spectroscopy [19]. From a characteristic EPR signature, they concluded the presence of a fully assembled, intact distal [Fe_4_S_4_]-cubane cluster with a His-to-Gln coordination, but could not rationalize the effect of this mutation on redox potentials and ET rates. According to our results, we expect a significant effect of the glutamine coordination to the distal [FeS]-cluster on ET rates. When glutamine coordinates the distal FeS-cluster as a deprotonated glutaminate, the absence of a π-electron system and the similarity of the amino acid side-chain size and charge to a cysteine lead us to hypothesize on an absent or significantly reduced ET from the distal FeS-cluster to an electron acceptor. In the uptake hydrogenase from *Nostoc punctiforme*, it was even possible to engineer a reverse electron flow by modifying the proximal FeS-cluster. The engineered enzyme was able to take up electrons via the distal [4Fe–4S]-cluster and transfer them to the [NiFe]-centre [19]. This is clearly also associated with the glutamine-to-histidine modified [FeS]_d_-cluster motif.

## 3. Materials and Methods

The protein crystal structure of the [NiFe]-hydrogenase from *D. fructosovorans* (pdb entry 1YWQ) was used. All calculations were performed using Turbomole v6.6 (Turbomole GmbH, Karlsruhe, Germany) [20,21]. The hybrid B3LYP [22,23,24] exchange correlation functional was used with a triple-zeta plus polarization functions (TZVP) basis set [25]. This combination was already shown to give ET parameters in good agreement with experiment. It was shown that B3LYP and PBE0 functionals give an identical ordering of spin states in cubanoid iron–sulfur clusters and identical assignment of oxidation states for each metal atom [26].

The calculations were carried out at the spin-unrestricted density functional theory level. Molecular orbitals were obtained from single-point energy calculations at the optimized structures. The matrix of overlap integrals of contracted atomic Gaussian basis functions was printed in separate supramolecular calculations of the donor plus the acceptor using the *$intdebug* option of TURBOMOLE. MOs and overlap matrices *S_mn_* used in subsequent calculations of *S* and *V_DA_* were calculated using an in-house developed code.

The reorganization energy was computed for separate donor models of the distal iron–sulfur cluster and coronene and circumcoronene as electron acceptors. All calculations for *λ* were structural optimizations on individual cluster models of the iron–sulfur cluster and coronene/circumcoronene as acceptor in different redox states. The oxidized iron–sulfur cluster is an EPR-silent S = 2 spin state of a [4Fe–4S]^0^ core made up of 2 Fe(II) and 2 Fe(III) atoms. The reduced form is a paramagnetic *S* = 1/2 spin state with a [4Fe–4S]^+^ core (3 Fe(II) and 1 Fe(III) atom). The redox states of the iron–sulfur clusters were elucidated by redox titrations and EPR monitoring [27].

Differences in reorganization energies between oxidized and reduced states originate from parabolic diabatic curves with different curvatures, for a discussion see for example [28].

Optimizations were performed *in vacuo* as well as in solution using the conductor-like screening model (COSMO [29]) to allow discriminating between internal and solvent reorganization energies (see above). Rates of electron transfer were calculated using the Marcus equation between an anti-ferromagnetically coupled *S* = 1/2 distal FeS-cluster model and a graphite electron acceptor model (see above).

## 4. Conclusions

[NiFe]-hydrogenase enzymes provide spatially separate pathways for proton and electron transfer when oxidizing molecular hydrogen. The large subunit, which also harbors the active site, is responsible for disposal of product protons along a conserved glutamate residue. The generated electrons flow along a chain of FeS-clusters in the small subunit towards the electron acceptor, either a cytochrome or an electrode surface. The rate of electron transfer from the distal FeS-cluster to an anode in an enzymatic fuel cell is mediated by a non-standard (His)(Cys)_3_ coordination. The calculated rates of electron transfer by use of the Marcus equation are in excellent agreement with experiment. A histidine-to-cysteine mutation in the first coordination sphere reduces the rate of electron transfer by three orders of magnitude. Since the reorganization energy is unchanged, the difference in *k_ET_* is resulting from a change in electronic donor-acceptor coupling. The insight gained into this electronic coupling between amino acid sidechain properties and electron transfer rates allows subsequent protein engineering to carefully modulate rates and routes of electron transfer from an enzyme to an electrode, in order to produce electric energy from dihydrogen.

## Figures and Tables

**Table 1:** Calculated inner-sphere reorganization energies for distal [FeS]-cluster models.

Distal FeS-Cluster Model	Redox State	*λ_i_* (eV)
([Fe_4_S_4_](Cys)_3_(His))^−2/−1^	ox	0.18
	red	0.32
([Fe_4_S_4_](Cys)_4_)^−3/−2^	ox	0.19 [10]
	red	0.30 [10]

**Table 2:** Calculated outer-sphere solvent reorganization energies *λ_S_* for electron donor and acceptor models in a conductor-like screening model (COSMO) (*ε* = 78) dielectric (in eV).

Donor/Acceptor for ET	Cluster Composition	Redox State	*λ_S_* (eV)
Donor [FeS]_d_-Cluster Model	([Fe_4_S_4_](Cys)_3_(His))^−2/−1^	ox/red	0.38/0.18
	([Fe_4_S_4_](Cys)_4_)^−3/−2^	ox/red	0.30/0.19
Acceptor Graphite Model	Coronene C_24_H_12_^−1/0^	ox/red	0.09/0.07

**Table 3:** Calculated direct electron transfer (DET) rates from the distal iron–sulfur cluster to a graphite electrode surface. Distances from distal iron–sulfur cluster to graphite surface (*Z*), overlap integrals of donor and acceptor orbitals (*S*), electronic coupling matrices (*V_DA_*), and calculated rates of electron transfer (ET) (*k_ET_*).

Distal FeS-Cluster Model	*Z* (Å)	*S*	*V_DA_* (cm^−1^)	*k_ET_* (s^−1^)
[Fe_4_S_4_](Cys)_3_(His)	12.3	5.3 × 10^−6^	4.4 × 10^−2^	5.1 × 10^3^
[Fe_4_S_4_](Cys)_4_	12.3	1.1 × 10^−7^	1.5 × 10^−3^	6.0

**Table 4:** Calculated through-space electron transfer rates from the distal iron–sulfur cluster to a graphite electrode surface. Distances from distal iron–sulfur cluster to graphite surface (*Z*), overlap integrals of donor and acceptor frontier orbitals (*S*), electronic coupling matrices (*V_DA_*), and calculated rates of ET (*k_ET_*).

Distal FeS-Cluster Model	*Z* (Å)	*S*	*V_DA_* (cm^−1^)	*k_ET_* (s^−1^)	Experiment [16] (s^−1^)
[Fe_4_S_4_](Cys)_3_(His)	15.3	8.2 × 10^-6^	2.9 × 10^-2^	2.2 × 10^3^	1–3.5 × 10^3^
[Fe_4_S_4_](Cys)_4_	15.3	2.9 × 10^-6^	1.1 × 10^-2^	3.6 × 10^2^	>40
